# The relationship between hypertensive disorders in pregnancy and endometriosis: a systematic review and meta-analysis

**DOI:** 10.1186/s12978-024-01833-x

**Published:** 2024-06-26

**Authors:** Foruzan Sharifipour, Zaynab Mohaghegh, Zahra Javanbakht, Shahla Faal Siahkal, Faeze Azizi

**Affiliations:** 1 Clinical Research Development Center, Motazedi Hospital, Kermanshah University of Medical Sciences, Kermanshah, Iran; 2 Family Health Department, Tehran University of Medical Sciences, Tehran, Iran; 3 Obstetrics and Gynecology Department, Motazedi Hospital, Kermanshah University of Medical Sciences, Kermanshah, Iran; 4 Department of Midwifery, Marand Branch, Islamic Azad University, Marand, Iran; 5 Student Research Committee, School of Nursing and Midwifery, Kermanshah University of Medical Sciences, Kermanshah, Iran

**Keywords:** Endometriosis, Gestational hypertension, Preeclampsia, Systematic review

## Abstract

**Background:**

Endometriosis is a chronic and debilitating disease that can affect the entire reproductive life course of women, with potential adverse effects on pregnancy. The aim of the present study is to investigate the association between hypertensive disorders in pregnancy and endometriosis.

**Method:**

Relevant articles were searched from the Cochrane Library, PubMed, Scopus and Web of Science from inception up to December 2023. The full-text observational studies published in English that had a confirmed diagnosis of endometriosis were included. The case group included pregnant women diagnosed with endometriosis at any stage, while the control group consisted of pregnant women who had not been previously diagnosed with endometriosis. Two authors extracted and analyzed the data independently. Disagreements were reconciled by reviewing the full text by a third author. Endnote X9 was used for screening and data extraction. We used fixed and random effects models in Review Manager 5.3 to analyze the pooled data. The quality of the included studies was assessed using the Downs and Black checklist.

**Results:**

Out of the 9863 articles reviewed, 23 were selected for meta-analysis. According to the results of this study, there was an association between endometriosis and gestational hypertension (OR = 1.11, 95% CI: 1.06, 1.16; *I*^2^ = 45%, *P* < 0.00001; *N* = 8), pre-eclampsia (OR = 1.26, 95% CI: 1.18, 1.36; *I*^2^ = 37%, *P* < 0.00001; *N* = 12), and hypertensive disorders in pregnancy (OR = 1.13, 95% CI: 1.06, 1.21; *I*^2^ = 8%, *P* = 0.0001; *N* = 8).

**Conclusions:**

This study confirmed that endometriosis may elevate the risk of developing gestational hypertensive disorders. Raising awareness of this issue will help to identify effective strategies for screening and early diagnosis of hypertensive disorders in pregnancy.

## Introduction

Endometriosis is a chronic gynecological disease characterized by the presence and growth of estrogen-dependent endometrial structures outside the uterine cavity, particularly on ovaries, fallopian tubes, pelvic peritoneum, and uterosacral ligaments [1]. Pelvic pain and infertility are the most common symptoms of affected women, occurring in 10–15% of women of reproductive age [2]. In the diagnosis of endometriosis based on ESHER guidelines, the presence of clinical symptoms, along with symptoms detected in clinical examinations and imaging (MRI and ultrasound) are used, and in case of suspicion of peritoneal endometriosis, laparoscopy is used for definitive diagnosis along with histological examination [3]. Clinical symptoms of endometriosis include abnormal bowel movements, intestinal dysfunction, dyspareunia, lower abdominal pain, severe dysmenorrhea, and infertility [4]. On the other hand, the prevalence of psychological disorders such as anxiety and depression is reported high in affected women [5]. Surgical excision of the lesion is a common treatment method that can alleviate pain and greatly enhance quality of life [6]. Of course, in some cases, recurrence of the disease has been reported [7].

Hypertensive disorders of pregnancy (chronic hypertension, gestational hypertension, and pre-eclampsia) are so prevalent throughout the world and can lead to serious consequences for both the mother and the baby [8]. The global prevalence of this disorder is almost 116 per 100,000 women of reproductive age. However, it varies depending on the region [9]. Current risk factors for hypertensive disorders include primigravida, increasing age, pre-pregnancy obesity, twin or multiple pregnancy, and some chronic diseases like polycystic ovarian syndrome (PCOs), overt diabetes, chronic kidney disease (CKD), and autoimmune disease [10]. Although the relationship between endometriosis and hypertensive disorders is not clearly defined, these two conditions seem to follow the same pathophysiological mechanisms.

Endometriosis is known as an immunological and chronic inflammatory disease [11]. It has been shown that the concentration of immunological and inflammatory factors such as macrophages, natural killer cells (NK cells), cytokines, B and T lymphocytes, growth factors, and angiogenesis stimulants is higher in women with endometriosis [10, 12], which can impede maternal and fetal adaptation with the normal changes of pregnancy. Additionally, a variety of immune cells and mediators have been associated with the onset of preeclampsia, a condition in which oxidative stress is linked to activation of the maternal inflammatory response. Immune cells such as regulatory T cells, macrophages, NK cells, and neutrophils are known to contribute significantly to the pathology of preeclampsia [13]. The interference caused by inflammatory and immunological responses can have a detrimental effect on trophoblast invasion and placental implantation that occurs by affecting the decidua and the placenta, which are crucial components of the process [14]. Defects in placental invasion or inappropriate remodeling of uterine spiral arteries can lead to blood pressure disorders in pregnancy [15]. Therefore, it seems that inflammatory and immunological factors play a role in pathogenesis of these two conditions and that they can affect each other.

However, the evidence regarding the link between hypertensive disorders of pregnancy and endometriosis seems to be conflicting. While some studies have shown a significant association between the two [16], others have suggested the opposite [17]. Moreover, some have found no relationship between these two conditions [18]. This disparity in results may stem from differences in study methodologies, sample sizes, endometriosis severity and location, or the presence of selection bias. Therefore, it is important to elucidate the role of endometriosis as a predictor of subsequent hypertensive disorders in patients with endometriosis who conceived spontaneously. The aim of the current systematic review was to investigate the potential link between hypertensive disorders during pregnancy and endometriosis.

## Material and methods

### Study protocol

This systematic review and meta-analysis of observational studies was conducted in accordance with the Preferred Reporting Items for Systematic Reviews and Meta-Analysis (PRISMA) guidelines [19]. The protocol of this systematic review was registered in PROSPERO (Ref No: CRD 42024498946).

### Search strategy

In this review, we included studies published in databases from inception up to December 2023. Systematic searches were performed on PubMed, Scopus, Cochrane Library, and Web of Science using MeSH keywords and terms. The keywords used were “Endometriosis” along with “Preeclampsia” and “Hypertension of pregnancy”.

### Inclusion and exclusion criteria

The selection of relevant studies was according to the following inclusion criteria: observational studies (case–control, cross-sectional, or cohort) and studies published in English. Studies written in local languages or with qualitative, review, and interventional designs, case report studies, congress presentations, or study protocols were excluded from this review. Furthermore, studies without a clear statement about the diagnosis of endometriosis, those lacking data on exposure or outcome, and those whose full text was not available were also excluded.

All the included studies had a confirmed diagnosis of endometriosis either by the presence of lesions during surgery (with or without histological confirmation), by imaging modality, or by International Classification of Disease (ICD)-coded medical records in women who conceived spontaneously. Due to the higher risk of obstetric complications such as pregnancy-induced hypertension and preeclampsia associated with pregnancies conceived through ART [20], we excluded studies on these topics in order to eliminate their potential impact on the relationship between endometriosis and hypertensive disorders. Diagnosis of the gestational hypertension was defined as a systolic blood pressure of ≥ 140 mmHg or a diastolic blood pressure of ≥ 90 mmHg after 20 weeks of gestation or based on definition of International Classification of Diseases (ICD) 8, 9, 10 codes for gestational hypertensive disorders or etc.

### Study participants

The case group in this study included pregnant women diagnosed with endometriosis at any stage or severity, while the control group consisted of pregnant women who had not been previously diagnosed with endometriosis. All the studies included in the review involved only women who had conceived naturally. Women who had become pregnant using in vitro fertilization were excluded from the study.

### Types of outcome measures

Outcomes of this study were the hypertensive disorders of pregnancy including pre-eclampsia and gestational hypertension.

### Study selection and data extraction

FSH and SHF conducted a search on the databases and screened the titles and abstracts of the search results based on specific criteria. They independently extracted data from eligible full texts. In case of any discrepancies or conflicts, a third author was consulted to resolve the issue. Endnote X9 was used for screening and data extraction. A table was created for data extraction, and the following pieces of information were extracted: study author’s name, study location, study type, participants’ age, sample size of the control and case groups, definitions of PIH and endometriosis, and outcomes.

### Assessment of study quality

FSH and SHF evaluated the quality of the studies included in the research using the checklist of Downs and Black (1998). The checklist comprised of twenty-seven questions that evaluated various areas. It included ten questions for assessing reporting bias, three for assessing external validity, seven for evaluating internal validity, six for assessing selection bias, and one question for assessing the power of the study [21]. The total quality score was classified as follows: a score of less than 14 was considered poor, a score between 15 and 19 was considered fair, and a score more than 20 was considered good [22].

### Statistical analysis

We conducted a meta-analysis using Review Manager version 5.4 (RevMan 5.4; Cochrane Collaboration, Oxford, UK) and set the significance level at less than 0.05. Mean differences (MD) and 95% confidence intervals (95% CI) were used to compare variables between groups. We used a fixed-effect meta-analysis to combine the mean differences of each study and demonstrated effect sizes and 95% CI using forest plots. We measured heterogeneity using *I*^2^, where an *I*^2^ value of 0–50% indicated low or moderate heterogeneity, and *I*^2^ > 50% indicated substantial heterogeneity. We used the random effects model when *I*^2^ > 50%. We conducted a sensitivity analysis to investigate potential sources of heterogeneity in case there was statistically significant heterogeneity across studies. In the sensitivity analyses, we systematically excluded one study at a time to test the strength of uncertainty in the meta-analysis [23]. We also statistically evaluated potential publication biases using funnel plots and Begg’s and Egger’s tests using STATA [24]. A funnel plot was used to assess publication bias whenever there were more than ten studies in the meta-analysis [25].

## Results

### Study selection

**Fig. 1:**
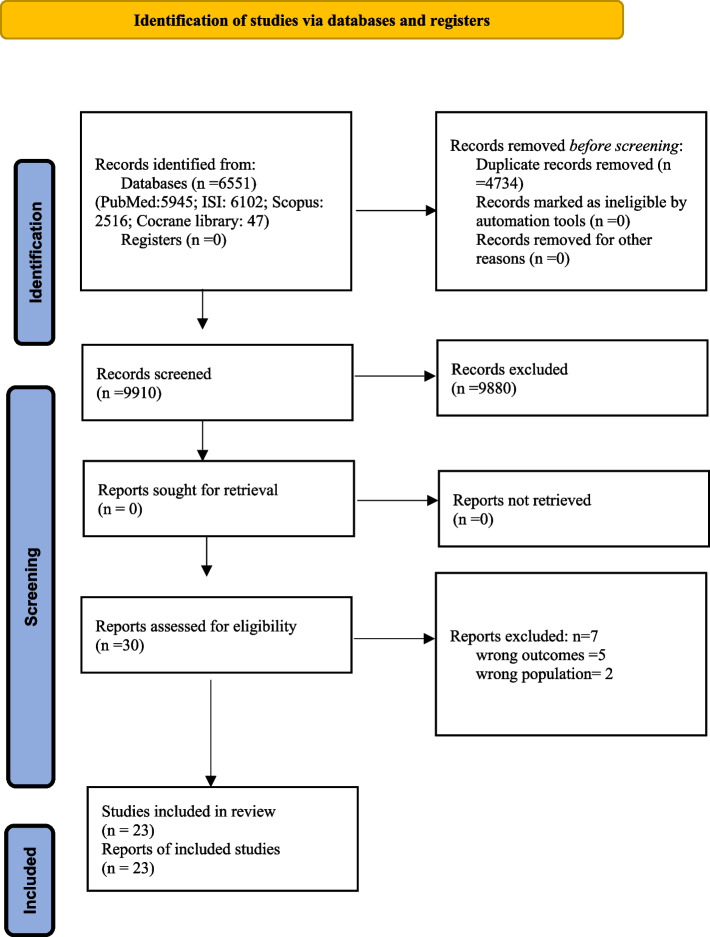
The flow diagram of the systematic review for selection of the studie

We obtained 14610 publications via the electronic search strategy (Web of Sciences: 6102; PubMed: 5945; Scopus; 2516; Cochrane Library: 47) from inception to 15 December 2023. Of these publications, 4734 duplicates were removed, and 9910 were subjected to title and abstract screening. Thirty articles were selected for eligibility at full-text review of which, 23 were eligible to be included in this review. Figure 1 shows the flowchart of the study.

### Study characteristics

Description of the studies is shown in Table 1. The reviewed observational studies included cohort studies (13 papers), case–control studies (7 papers), and longitudinal design (3 papers). As far as the country of origin of the studies was concerned, four were performed in Italy [26–29], three in Japan [30–32], three in China [6, 33, 34], three in Australia [18, 35, 36], two in the UK [17, 37], two in Denmark [38, 39], two in the USA [40, 41], one in France [42], one in Taiwan [10], one in Sweden [16], and one in Canada [43]. The number of participants in the studies varied from 40 to 1,429,585. In all studies, women of reproductive age were included. The diagnosis of endometriosis in the included studies was based on the results of laparoscopy, surgery, diagnosis code of International Classification of Diseases ICD 9—ICD10, or imaging. In this review, 133,941 women with endometriosis were compared with 8,932,888 healthy women in terms of hypertensive disorders of pregnancy. Table 2 shows the definitions of hypertensive disorders and endometriosis across all included studies.

**Table 1:** Characteristics of studies included in the systematic review

No	Study	Location	Study type	Age (y); mean ± SD		No. of participants		Gravidity of participants	Main outcomes: N (%)	
				**Case**	**Control**	**Case**	**Control**		**Case**	**Control**
1	Brosens et al. 2007 [17]	UK	Retrospective case–control study	32 (21– 44)	33 (20– 44)	245	274	Nulliparous and multiparous	Pre-eclampsia	
									2 (0.8%)	16 (5.8%)
2	Berlac et al. 2017 [38]	Denmark	National cohort study	31.4	30.4	11 739	615 533	Nulliparous and multiparous	Hypertension	
									404 (2.1%)	18984 (1.8%)
									Pre-eclampsia	
									588(3.0%)	23 625 (2.2%)
3	Conti et al. 2015 [26]	Italy	Cohort study	-	-	219	1331	Nulliparous and multiparous	Gestational hypertension	
									8 (3.7%)	77 (5.8%)
									Preeclampsia	
									5 (2.2%)	16 (1.2%)
4	Epelboin et al. 2021 [42]	France	Longitudinal study	31.7 ± 4.8	30.0 ± 5.3	31,101	4,083,732	Nulliparous and multiparous	Preeclampsia	
									679 (2.18%)	64,288 (1.57%)
5	Farland et al. 2019 [40]	USA	Prospective cohort study	29.1 ± 5.3	29.1 ± 5.3	8,875	187,847	Nulliparous and multiparous	Hypertensive disorders of pregnancy	
									541/5,665 (9.5%)	8,730/131,970 (6.6%)
6	Farland et al. 2022 [41]	USA	Cohort study	33.0 ± 4.03	34.54 ± 4.23	1,560	73,868	Nulliparous and multiparous	PIH/Pre-eclampsia	
									213 (13.7%)	9,440 (10.5%)
7	Gebremedhin et al. 2023 [35]	Australia	Population-based retrospective cohort study	15–49	15–49	19,476	893,271	Nulliparous and multiparous	Preeclampsia	
									1468 (7.5%)	54,098 (6.1%)
8	Glavind et al. 2017 [39]	Denmark	Danish cohort study	30–34	25–29	1,719	81,074	Nulliparous and multiparous	Pre-eclampsia	
									89 (5.18%)	3519 (4.34%)
9	Hadfield et al. (2009) [18]	Australia	Population-based, longitudinal study	31.4 ± 5.1	28.3 ± 5.7	3239	205 640	Nulliparous and multiparous	Gestational hypertension	
									352(10.9%)	23 186 (11.3%)
									Pre-eclampsia	
									103 (3.2%)	6564 (3.2%)
10	Harada et al. (2016) [30]	Japan	Prospective cohort study	15–45	15–45	330	8,856	Nulliparous and multiparous	Pre-eclampsia	
									8 (2.4%)	281(3.1%)
11	Ibiebele et al. 2022 [36]	Australia	Population-based cohort study	32.0 ± 5.1	29.7 ± 5.7	13 406	556922	Nulliparous and multiparous	Pregnancy hypertension	
									1378 (10.3%)	50 231 (9.0%)
12	Lin et al. 2015 [33]	China	Retrospective cohort study	32.8 ± 4.0	30.6 ± 3.5	249	249	Nulliparous—multiparous	Pregnancy-induced hypertension	
									9 (3.6%)	11 (4.4%)
13	Liu et al. 2023 [6]	China	Retrospective study	31.96 ± 4.38	31.75 ± 4.33	1026	2783	Nulliparous and multiparous	Gestational hypertension	
									19 (1.85%)	60 (2.16%)
									Preeclampsia	
									38 (3.70%)	61 (2.19%)
14	Mekaru et al. 2013 [31]	Japan	Retrospective analysis	33.0 ± 3.8	33.6 ± 4.1	49	59	Nulliparous and multiparous	Pregnancy-induced hypertension	
									6 (15%)	6 (12.5%)
15	Miura et al. 2019 [32]	Japan	Case–control study	34.2 ± 4.6	32.9 ± 5.2	80	2689	Nulliparous and multiparous	Hypertensive Disorders of Pregnancy	
									4 (5.0%)	187 (7.0%)
16	Pan et al. 2017 [10]	Taiwan	Population-based longitudinal cohort study	31.77 ± 5.76	31.77 ± 5.76	2578	10312	Nulliparous and multiparous	Gestational hypertension-preeclampsia	
									100 (3.88%)	168 (1.63%)
17	Saraswat et al. 2016 [37]	UK	National population-based cohort study	30.5 ± 5.2	27.2 ± 6.1	5375	8280	Nulliparous and multiparous	Hypertensive disorders	
									350 (8.3%)	452 (6.7%)
18	Stephansson et al. 2009 [16]	Sweden	Population-based longitudinal study	< 20-> 35	< 20- > 35	13 090	1 429 585	Nulliparous and multiparous	Pre-eclampsia	
									441 (3.37%)	41 377 (2.89%)
19	Porpora et al. 2020 [27]	Italy	Prospective cohort study	31(18–45)	29(18–42)	145	280	Nulliparous women	Pregnancy induced hypertension	
									7 (5%)	16 (6%)
									Preeclampsia	
									3 (2%)	2 (1%)
20	Scala et al. 2019 [28]	Italy	Retrospect analysis	30.2 (26.8–33)	30.3(27–33)	40	80	Nulliparous and multiparous	Preeclampsia	
									9 (7.5%)	6 (7.5%)
21	Uccella et al. 2019 [29]	Italy	Retrospective case–control study	34(22–45)	31(15–48)	118	1,690	Nulliparous women	Hypertension/preeclampsia	
									13 (11%)	99 (5.9%)
22	Velez et al. 2022 [43]	Canada	Population-based cohort study	32.95 ± 4.88	30.03 ± 5.6	19,099	768,350	Nulliparous and multiparous	Hypertensive disorder	
									1042 (5.5%)	37660 (4.9%)
23	Xie et al. 2023 [34]	China	Case–control study	30.96 ± 3.32	30.23 ± 2.98	188	188	Nulliparous and multiparous	Hypertensive disorder in pregnancy	
									10	11

**Table 2:** Definition of hypertensive disorders and endometriosis in the included studies

Study	Gravidity of participants	Definitions of PIH	Definitions of endometriosis
Brosens et al. 2007 [17]	Nulliparous and multiparous	Persistently raised blood pressure (140/90 mmHg) starting after the 20th week of gestation. Pre-eclampsia was defined as PIH with proteinuria (> 300 mg/24 h)	Laparoscopy
Berlac et al. 2017 [38]	Nulliparous and multiparous	International Classification of Diseases (ICD)-10 codes	women who underwent surgical interventions for their disease before pregnancy
Conti et al. 2015 [26]	Nulliparous and multiparous	Systolic blood pressure over 140 mmHg or diastolic blood pressure over 90 mmHg after 20 weeks of gestation. preeclampsia: hypertension developing after 20 weeks of gestation with proteinuria	pathology following surgical removal of the lesions
Epelboin et al. 2021 [42]	Nulliparous and multiparous	-	The diagnosis of endometriosis was recorded if reported in previous hospitalizations since 2008
Farland et al. 2019 [40]	Nulliparous and multiparous	self-report	Laparoscopy
Farland et al. 2022 [41]	Nulliparous and multiparous	ICD 9 and 10 codes	ICD9 and ICD10 codes
Gebremedhin et al. 2023 [35]	Nulliparous and multiparous	ICD-9/ICD-9-CM	ICD-9/10^th^ revision-Australian Modification)
Glavind et al. 2017 [39]	Nulliparous and multiparous	using the relevant ICD-8 and ICD-10 from the Danish National Patient Registry	laparoscopic surgery
Hadfield et al. (2009) [18]	Nuliparous and multiparous	The ICD-10 codes used to define	The ICD-10 codes used to define
Harada et al. (2016) [30]	Nuliparous and multiparous	as persistently raised blood pressure ≥ 140/90 mmHg, occurring after > 20 weeks Preeclampsia with severe features was defined as severe blood pressure elevation and severe proteinuria	Questionnaire
Ibiebele et al. 2022 [36]	Nuliparous and multiparous	-	Australian modification (ICD10-AM)
Lin et al. 2015 [33]	Nulliparous—multiparous	Elevated blood pressure ≥ 140/90 mmHg after 20 weeks of gestation Preeclampsia is gestational hypertension with proteinuria	confirmed histologically and visually at the surgical procedure
Liu et al. 2023 [6]	Nulliparous and multiparous	‘Hypertension in pregnancy’ was defined as a systolic blood pressure of ≥ 140 mmHg or a diastolic blood pressure of ≥ 90 mmHg. When measured with semiquantitative urine dipsticks, proteinuria of at least 1 + in the presence of hypertension, with no evidence of urinary tract infection, was considered significant	Diagnosis of endometriosis was done by laparoscopic examination, and the stage of endometriosis (was determined based on the revised American Society for Reproductive Medicine (rASRM) classification
Mekaru et al. 2013 [31]	Nulliparous and multiparous	-	laparoscopic evaluation
Miura et al. 2019 [32]	Nulliparous and multiparous	-	laparoscopy with histological confirmation
Pan et al. 2017 [10]	Nulliparous and multiparous	International Classification of Diseases, 9th Revision, Clinical Modification (ICD-9-CM)	Surgical assessment by laparoscopy or laparotomy
Saraswat et al. 2016 [37]	Nulliparous and multiparous	-	surgically confirmed
Stephansson et al. 2009 [16]	Nulliparous and multiparous	ICD-9 codes	-
Porpora et al. 2020 [27]	Nulliparous women	-	surgical/histological or clinical /instrumental diagnosis of endometriosis
Scala et al. 2019 [28]	Nulliparous and multiparous	Gestational hypertension and concomitant proteinuria	Ultrasonographic diagnosis of endometriosis
Uccella et al. 2019 [29]	Nulliparous women	-	Pervious surgery
Velez et al. 2022 [43]	Nulliparous and multiparous	-	surgery with a diagnosis code of International Classification of Diseases ICD 9–617 or ICD10-N80
Xie et al. 2023 [34]	Nulliparous and multiparous	Increase in blood pressure of ≥ 140/ 90 mmHg after 20 weeks of gestation. Preeclampsia is gestational hypertension with proteinuria	Histological examination

### Meta-analysis of outcomes

#### Gestational hypertension

**Fig. 2:**
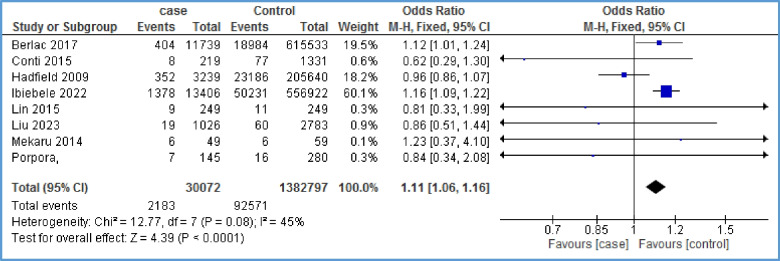
Forest plot showing the relationship between gestational hypertension and endometriosis between the two case and control group

The relationship between gestational hypertension and endometriosis was investigated in 8 studies [6, 18, 26, 27, 31, 33, 36, 38]. The evidence showed a positive and significant statistical relationship between the two mentioned variables (OR = 1.11, 95% CI: 1.06, 1.16; *I*^2^ = 45%, *P* < 0.0001; *N* = 8) (Fig. 2). Due to the limited number of papers on the relationship between gestational hypertension and endometriosis, it was not possible to generate a funnel plot.

### Pre-eclampsia

**Fig. 3:**
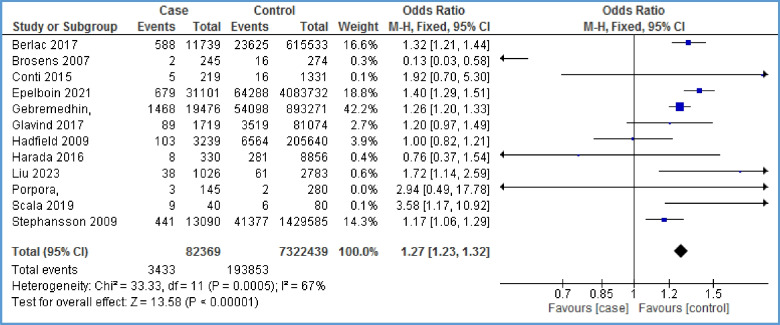
Forest plot showing the relationship between pre-eclampsia and endometriosis between the two case and control group

**Fig. 4:**
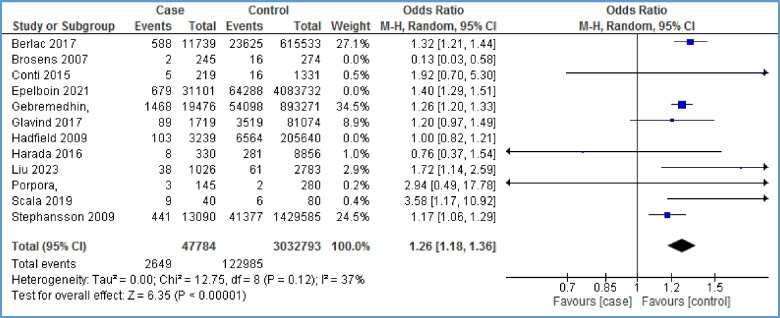
Forest plot of sensitivity analysis showing the relationship between pre-eclampsia and endometriosis between the two case and control group

**Fig. 5:**
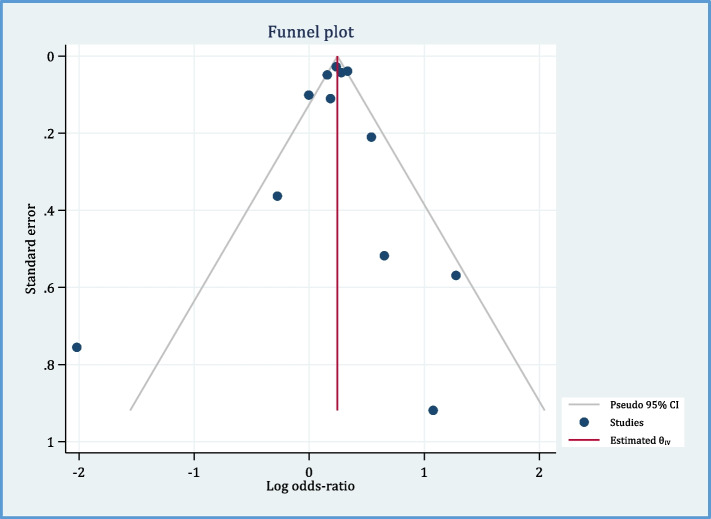
Funnell plot of included studies to assess the potential publication bias

Twelve papers reported the relationship between pre-eclampsia and endometriosis [6, 16–18, 26–28, 30, 35, 38, 39, 42]. As Fig. 3 shows, there is a positive relationship between pre-eclampsia and endometriosis (OR = 1.27, 95% CI: 1.23, 1.32; *I*^2^ = 67%, *P* < 0.00001; *N* = 12). Because of high heterogeneity, we performed sensitivity analysis. By removing the effect of three studies [17, 18, 42] on the overall results, heterogeneity reached 44%, and still, the evidence indicated a statistically significant positive relationship between endometriosis and preeclampsia (OR = 1.26, 95% CI: 1.18, 1.36; *I*^2^ = 37%, *P* < 0.00001) (Fig. 4). Based on this, the chance of developing pre-eclampsia in the case group is 1.26 times that of the control group. In other words, the chance of developing pre-eclampsia in the case group is 26% higher than that in the control group. The distribution of points in the funnel plot (Fig. 5) as well as the Egger test results in Table 3 show that there is no publication bias (*P*-value = 0.808).

**Table 3:** Egger test results for publication bias

	**Beta (SE)**	**Z**	***P*-value**
**Egger test for pre-eclampsia**	-0.11 (0.47)	-0.24	0.808

### Hypertensive disorders

**Fig. 6:**
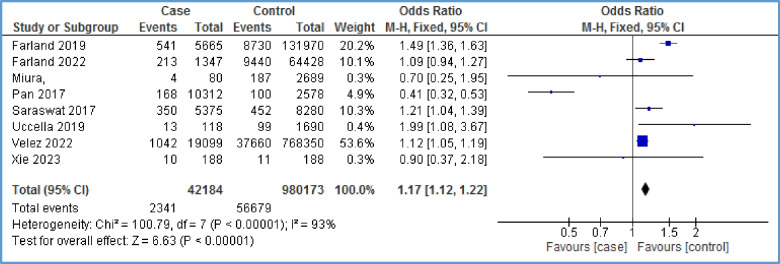
Forest plot showing the relationship between hypertensive disorders and endometriosis between the two case and control group

**Fig. 7:**
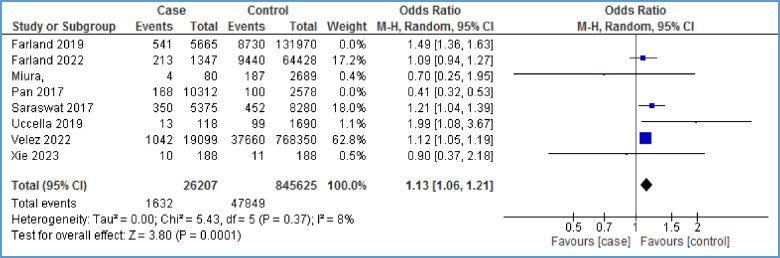
Forest plot of sensitivity analysis showing the relationship between hypertensive disorders and endometriosis between the two case and control group

Eight papers [10, 29, 32, 34, 37, 40, 41, 43] assessed the overall occurrence of hypertensive disorders (combined gestational hypertension-preeclampsia) in women affected with endometriosis. The meta-analysis showed a statistically significant relationship between hypertensive disorders and endometriosis with high heterogeneity (OR = 1.17, 95% CI: 1.12, 1.22; *I*^2^ = 93%, *P* < 0.00001; *N* = 8) (Fig. 6). To reduce heterogeneity, we omitted the effect of two papers [10, 40] on the overall results. Heterogeneity reached eight percent, and a statistically significant relationship between the two variables was identified (OR = 1.13, 95% CI: 1.06, 1.21; *I*^2^ = 8%, *P* = 0.0001) (Fig. 7). In other words, the chance of developing hypertensive disorder in the case group is 13% higher than that in the control group. A funnel plot could not be generated due to the limited number of papers on the relationship between hypertensive disorders and endometriosis.

### Assessment of the risk of bias within studies

The quality assessment of the included studies is shown in Table 4. The median total quality score was 16 which represented moderate quality.

**Table 4:** Quality assessment of the articles reviewed

Study ID (Author, Year)	Clarity	External validity	Internal validity		Power	Total score
			**Bias**	**Confounding**		
Brosens et al. 2007 [17]	9	3	3	2	0	17
Berlac et al. 2017 [38]	7	3	3	3	0	16
Conti et al. 2015 [26]	7	2	3	2	0	14
Epelboin et al. 2021 [42]	9	3	3	3	0	18
Farland et al. 2019 [40]	9	3	4	3	0	19
Farland et al. 2022 [41]	9	3	4	3	0	19
Gebremedhin et al. 2023 [35]	8	3	3	3	0	17
Glavind et al. 2017 [39]	8	3	3	4	0	18
Hadfield et al. 2009 [18]	7	3	3	2	1	16
Harada et al. 2016 [30]	8	3	3	3	0	17
Ibiebele et al. 2022 [36]	9	3	3	3	1	19
Lin et al. 2015 [33]	9	1	3	2	0	15
Liu et al. 2023 [6]	7	3	4	2	0	16
Mekaru et al. 2013 [31]	7	1	4	2	1	15
Miura et al. 2019 [32]	9	3	4	3	0	19
Pan et al. 2017 [10]	9	3	4	3	1	21
Saraswat et al. 2016 [37]	9	3	4	3	1	21
Stephansson et al. 2009 [16]	9	3	4	3	1	20
Porpora et al. 2020 [27]	7	1	4	2	0	14
Scala et al. 2019 [28]	9	0	3	2	0	15
Uccella et al. 2019 [29]	5	0	3	1	0	9
Velez et al. 2022 [43]	9	3	3	3	1	19
Xie et al. 2023 [34]	8	2	4	3	1	18
Mean range						16

## Discussion

This systematic review aimed to evaluate the correlation between hypertensive disorders in pregnancy and endometriosis**.** We included 23 observational studies which had a moderate quality score on average. The pooled evidence in this meta-analysis showed that the odds of gestational hypertension and preeclampsia were higher in women with endometriosis when compared to those without endometriosis. Endometriosis is an important cause of infertility. Pathophysiological speaking, it is expected to affect pregnancy outcomes [14]. Hormonal and inflammatory changes that occur in pregnancy are essential to ensure proper decidualization and placentation. In addition, these changes are also necessary to maintain pregnancy and active labor at term. Similarly, in endometriosis, there are hormonal changes and inflammatory factors that can overlap with pregnancy changes, ultimately causing disruption in pregnancy processes [44]. Cytokines, proteases, and matrix metalloproteinases play a major role in proper decidualization, which is necessary for successful blastocyst implantation. In endometriosis, inflammatory pathways that are regulated by decidua cells may be changed, which could lead to impaired proper trophoblast invasion and implantation [44]. Studies conducted on the relationship between endometriosis and hypertension disorders have yielded conflicting results. Similar to our findings, a systematic review by Breintoft et al. (2021) showed that endometriosis due to placental dysfunction is associated with an increased risk of adverse pregnancy outcomes including gestational hypertension and preeclampsia [45]. Although our study focused on women who conceived spontaneously, the population in Breintoft et al. consisted of all women who conceived with ART or spontaneously. Also, the number of included studies was small in the mentioned study.

A large population-based cohort study confirmed that there is a higher risk of preeclampsia in women with endometriosis compared to those without endometriosis [35]. It included a very large sample size, and its results support a significant association of endometriosis with an increased risk of preeclampsia and other outcomes including placenta previa and preterm birth. However, the results of a systematic review including more than one million women showed that endometriosis had no relationship with gestational hypertension or preeclampsia [46]. This is probably due to the limitations noted in that study, namely a) inconsistently adjusted confounding factors that applied among the multiple sets of data and b) diagnosis and management of pregnancy complications that could differ across the studies. In addition, in the mentioned review, the participants were women who had become pregnant after in vitro fertilization (IVF), but our study included women who had become pregnant spontaneously. Conversely, a cohort study involving 787,449 women with singleton pregnancies showed that endometriosis was associated with an increased risk of hypertensive disorders during pregnancy [43]. This finding may be explained by the fact that in women with endometriosis, changes in cytokines and thicker junctional zones of the myometrium cause inappropriate trophoblast invasion [44, 47]. Since the conversion of spiral arteries in the myometrial junctional zone is a necessary process for the formation of normal placenta, various characteristics of the junctional zone of endometriosis patients can cause abnormal placental function and thus increase the risk of pregnancy-induced hypertension disorders [47, 48].

This study has a significant strength because of the large number of studies reviewed and the large number of participants recruited, which increases the reliability of the conclusions. The accuracy of data was improved due to the absence of publication bias. Also, the diagnosis of endometriosis was confirmed in most cases using surgery and laparoscopy. To maintain consistency in the study results, we only included women who conceived naturally and excluded those who conceived through IVF.

Despite these strengths, this study had a number of limitations. Unfortunately, there was insufficient data in most studies to perform subgroup analysis based on endometriosis extension, clinical severity, duration of the illness, staging, and women’s age and parity, which could be considered as confounding factors. Additionally, about 50% of pregnant women with ovarian or deep endometriosis may be unaware of their condition [49]. As a result, there could be a significant number of women with endometriosis who are misdiagnosed due to lack of awareness about their condition, potentially impacting research results. It is important to note that adenomyosis, a condition related to endometriosis where the endometrium invades the myometrium, was not taken into account in this review. In addition, the study with the greatest significance in this meta-analysis was the one conducted by Ibiebele et al. (2022) [36], which established a strong and positive relationship between gestational hypertension and endometriosis. Other studies included in the analysis did not demonstrate a significant relationship between the two conditions. Therefore, more high-quality studies are needed to prove the relationship between these two medical conditions.

## Conclusion and recommendations

Our results showed that the odds of gestational hypertension and preeclampsia were higher in women with endometriosis compared to those without endometriosis. This finding help physicians to apply effective strategies for the screening and early diagnosis of hypertensive disorders in pregnancy, which could reduce the risk of maternal and fetal morbidity during pregnancy. However, we recommend that more high-quality studies be conducted to prove the relationship between gestational hypertension and endometriosis. Also, there is a need to conduct longitudinal observational studies to investigate the effect of endometriosis on hypertensive disorders based on the severity, staging, and location of endometriosis. The effect of endometriosis on spontaneous versus induced pregnancies with assisted reproductive methods should also be compared and examined.

## Data Availability

No datasets were generated or analysed during the current study.

## Abbreviations

PCOs: Polycystic Ovarian Syndrome
CKD: Chronic Kidney Disease
IUGR: Intrauterine Growth Restriction
LBW: Low Birth Weight
PRISMA: Preferred Reporting Items for Systematic Reviews and Meta-Analysis
ICD: International Classification of Disease
MD: Mean differences
CI: Confidence intervals
IVF: In vitro Fertilization
NK cells: Natural Killer cells
